# High Temperature and Salinity Enhance Soil Nitrogen Mineralization in a Tidal Freshwater Marsh

**DOI:** 10.1371/journal.pone.0095011

**Published:** 2014-04-14

**Authors:** Haifeng Gao, Junhong Bai, Xinhua He, Qingqing Zhao, Qiongqiong Lu, Junjing Wang

**Affiliations:** 1 State Key Laboratory of Water Environment Simulation, School of Environment, Beijing Normal University, Beijing, China; 2 NSW Department of Primary Industries, West Pennant Hills, New South Wales, Australia; Beijing Forestry University, China

## Abstract

Soil nitrogen (N) mineralization in wetlands is sensitive to various environmental factors. To compare the effects of salinity and temperature on N mineralization, wetland soils from a tidal freshwater marsh locating in the Yellow River Delta was incubated over a 48-d anaerobic incubation period under four salinity concentrations (0, 10, 20 and 35‰) and four temperature levels (10, 20, 30 and 40°C). The results suggested that accumulated ammonium nitrogen (NH_4_^+^-N) increased with increasing incubation time under all salinity concentrations. Higher temperatures and salinities significantly enhanced soil N mineralization except for a short-term (≈10 days) inhibiting effect found under 35‰ salinity. The incubation time, temperature, salinity and their interactions exhibited significant effects on N mineralization (*P*<0.001) except the interactive effect of salinity and temperature (*P*>0.05), while temperature exhibited the greatest effect (*P*<0.001). Meanwhile, N mineralization processes were simulated using both an effective accumulated temperature model and a one-pool model. Both models fit well with the simulation of soil N mineralization process in the coastal freshwater wetlands under a range of 30 to 40°C (*R^2^* = 0.88–0.99, *P*<0.01). Our results indicated that an enhanced NH_4_^+^-N release with increasing temperature and salinity deriving from the projected global warming could have profound effects on nutrient cycling in coastal wetland ecosystems.

## Introduction

Nitrogen (N) is the most limiting nutrient in flooded soils [1]–[3], especially in coastal wetland ecosystems [4], [5]. Studies have shown that N availability greatly affects plant growth, diversity and possible successional changes [6]–[8]. Organic N transformed into inorganic N via N mineralization primarily determines the soil N availability [2], [9], which is also closely related to greenhouse gas emissions (i.e., N_2_O, NO, and other forms). Therefore, a better understanding of N mineralization processes is important for improving the sustainability of N limiting ecosystems while reducing the risk of atmospheric pollution [10]–[12].

Nitrogen mineralization in wetlands is sensitive to various environmental factors, including air temperature, water tables, flooding periods and soil properties [3], [6], [13]–[15]. Numerous studies have demonstrated that soil temperature is a primary environmental factor that influences N mineralization processes in wetland soils [2], [3], [16]. For instance, Noe et al. [17] reported that N mineralization fluxes and turnover rates in continuously freshwater tidal forests to salt impacted tidal forest to oligohaline marsh along the Waccamaw river and Savannah river in the US were generally lower in March and December compared to June and September due to differences in microbial activity as affected by seasonal variation of soil temperature. Gilliam et al. [18] suggested that mild freezing might inhibit N mineralizing microbes and stimulate N immobilizing groups. However, Zhang et al. [19] demonstrated that mild freezing had few effects on N mineralization, while Gao et al. [2] observed higher N mineralization rates in winter than in summer in a seasonal flooding wetland. Therefore, a better understanding of how variation of air temperature could affect N mineralization is urgently needed since the potential increase of the earth's surface temperature could be 1.8–4.0°C at the end of this century [20].

Salinity is another important factor that influences N mineralization in coastal wetlands [2], [6], [21]. Specifically, alluvial soils are typically subjected to seepage or flooding during high tides and coastal storms. Moreover, the poor drainage from high groundwater tables contributes to salt accumulation in surface soils [2], [21]. However, studies have shown conflicting effects (i.e., inhibiting or improving) of salinity on nitrogen mineralization in wetlands [6], [17], [21], [22]. For example, Pathak and Rao [23] suggested that N mineralization decreased with increasing salinity and was biologically mediated at higher salinity levels. Gao et al. [2] found a slight increase in the net N mineralization rate in tidal salt marshes after freshwater input. However, Chen and Twilley [22] reported that relative N mineralization of mangrove soils decreased exponentially with increasing distance from the mouth of the Shark River estuary in the USA or decreasing salinity. More recently, Noe et al. [17] demonstrated that salinification increased net N mineralization fluxes in tidal freshwater wetland soils along the floodplains of both the Waccamaw and Savannah rivers in the USA. However, these studies have primarily relied on sites positioned along salinity gradients. This configuration may not be sufficient to identify the effects of salinity on N mineralization due to the different environmental conditions at these sites.

Studies on the salinification effects on nutrient biogeochemistry in tidal freshwater wetlands are limited even though these wetlands are sensitive to sea level rise and increased salinity due to their location between non-tidal freshwater rivers and tidal saline estuaries [17], [24]. As most tidal freshwater marshes may be substantially disturbed by the conversion of freshwater marshes to brackish marshes caused by accelerated sea-level rise effects [25], [26], changing salinity may become a dominant factor influencing ecological processes in tidal freshwater marshes in coastal regions. However, few studies have simultaneously examined the effects of temperature plus salinity caused by global warming in N-limiting tidal freshwater wetlands. Therefore, the primary objectives of this study were to (1) simulate N mineralization processes for different temperatures and soil salinity levels in a tidal freshwater wetland of the Yellow River Delta and (2) investigate the effects of temperature, salinity and their interactions on N mineralization rates.

## Materials and Methods

### Ethics statement

Our study area is located in the Yellow River Delta wetland nature preserve, which is owned by the Chinese government. Specific permits are required for conducting research in the preserve. However, our sampling sites were not located in any strictly protected areas containing endangered or protected species.

### Site description

The study area (37°35′–38°12′ N,118°33′–119°20′ E) is located in the estuary of the Yellow River Delta Wetland Nature Preserve, Shandong, China. This area has a typical monsoon (warm temperate) climate with an annual mean temperature of 11.9°C; the highest and lowest temperatures (39.7°C and −23.3°C, respectively) occur in July and January, respectively. Moreover, the annual mean precipitation is 640 mm (70% of the precipitation falls between June and August). Typical freshwater wetlands dominated by *P. australis* were selected. These sites were 150 m away from the southern riverbank along the lower reach of the Yellow River, where the soil is Eutric Fluvisol [27] with >85% of silt. The study site has a relatively higher topography compared with the floodplains near the river and can be flooded by the Yellow River after the annual flow-sediment regulation in late June. However, this area is likely to be flooded with seawater in the future due to potential increases in sea level.

### Soil sampling

Plant root system mainly distributed in upper soils, though the rhizome of *P. australis* could reach to more than 70cm soil depth. In general, top soils exhibited higher N contents and are more sensitive to environmental changes (e.g. temperature, hydrological changes) compared to deeper soils [4], [28]. Moreover, top soils (0–20 cm) in five sampling sites with five replicates were randomly collected from the freshwater marsh (*P. australis*) in September 2008 and transported to the laboratory at 4°C with an ice cooler. The soils were sieved through a 2 mm nylon sieve to remove recognizable plant litters, coarse roots, and stones and subsequently divided into two portions. One portion was stored at 4 °C for incubation, soil moisture, and initial NH_4_^+^-N and NO_3_^−^-N analyses. The other portion was air-dried for three weeks and subsequently ground and sieved (0.15 mm) for physical and chemical analyses (Table 1). Five additional soil cores (100 cm^3^) were collected to determine the soil bulk density.

**Table 1:** Physico-chemical properties of the tested soil.

	Average	SD
Moisture (%)	21.50	0.43
Bulk density (g cm^−3^)	1.79	0.05
Silt+Sand (%)	98.19	1.10
Clay (%)	1.79	1.10
Salinity (‰)	0.53	0.21
pH value	8.38	0.16
SOM (g kg^−1^)	6.47	1.92
Total N (g kg^−1^)	0.38	0.05
Total P (g kg^−1^)	0.65	0.02
C:N ratio	9.88	1.48
NH_4_^+^-N (mg kg^−1^)	1.46	0.57
NO_3_^−^-N (mg kg^−1^)	1.82	0.63

SD: Standard deviation.

### Incubation and chemical analysis

Every 20-g soil sample (oven-dried adjusted) was submerged with 15 ml of adjusted sea salt solution at the salinities 0‰, 10‰, 20‰ or 35‰ [29], [30] in 50 ml glass tubes for the salinity treatment experiments. The sea salt contained 55.03% Cl^−^, 30.59% Na^+^, 7.68% SO_4_^2−^, 3.68% Mg^2+^, 1.18% Ca^2+^ and 1.11% K^+^ [31]. The highest salinity (35‰), which is similar to seawater at study site, was used simulate a seawater flooding intrusion caused by future sea level rise. The 10 and 20‰ salinity gradients represented mixed river and seawater flooding situations due to the location between non-tidal freshwater rivers and tidal saline estuaries [17], [24]. Deionized water was used for the 0‰ salinity, which was used as a control scenario. Alcohol-wetted burning cotton was stretched into the tube for approximately two or three seconds. Then, the cotton was slowly pulled out while the flame shrank until extinction. The oxygen above the water surface in the tube was completely consumed through combustion. Immediately thereafter, the tubes were tightly capped with rubber bungs to prevent aeration. Nitrification could be inhibited in high moisture environments, especially in submerged conditions [28], [30]–[32]. Therefore, ammonium ought to reflect N mineralization [33], [34].

Each salinity treatment had 84 replicates, which were divided into four incubation groups. The groups remained in the dark for 48 days in the constant temperature incubators at 10, 20, 30 or 40°C. A pilot experiment observed the balance of the amount of mineralized N over 48-d incubation under higher temperature ranges (20°C, 30°C and 40°C) based on the potential mineralized N of the first-order reaction kinetics model. No obvious changes in N mineralization rates at 10°C were observed over a 48-d incubation period. We designated 48 days as our incubation period. Generally, these temperatures represented the mean seasonal air temperatures at the study site. Three tubes from each salt treatment and for each temperature were randomly chosen at 2, 5, 9, 14, 21, 35, and 48 days for analysis.

Soil NH_4_^+^-N was extracted with 2 M KCl (soil:solution = 1∶2.5). Extracts were mixed for 1 hour on a longitudinal shaker and filtered through #42 Whatman filter paper and stored at 4°C to determine NH_4_^+^-N with an Astoria Analyzer 300 system (Astoria Pacific International, Taiwan). Soil organic matter (SOM) was determined according to Walkley and Black [35]. Total carbon (TC) and total nitrogen (TN) were measured using an elemental analyzer (Vario El, Elementar Co., Germany). The initial soil nitrate nitrogen (NO_3_^−^-N) and ammonium nitrogen (NH_4_^+^-N) were determined in the 2 M KCl extracts with an Astoria Analyzer 300 system (Astoria Pacific International, Taiwan). Soil pH (H_2_O) was measured using a pH meter (soil: water = 1∶5). Water content was evaluated by drying the soil to a constant weight at 105°C for 24 h in an oven.

### Nitrogen mineralization models

Nitrogen mineralization was estimated using an effective accumulated temperature model (temperature model) [33], [36] and a first-order reaction kinetic model (one-pool model) [37]. The temperature model is described by
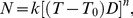
(1)where *N* is the mineralized soil organic N (mg kg^−1^), *k* is the constant mineralization rate, *T_0_* is the base point temperature (i.e., 15°C, below which microorganism activity is weak and *N* mineralization may be trivial), *D* is the incubation time at temperature *T* (day), and n is a mineralization constant.

The one-pool model is given by
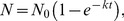
(2)where *k* is the instantaneous release rate, *N_o_* is the potential mineralized N as 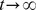 (mg kg^−1^), and *t* is the incubation time (day).

The active N fraction (ANF) in the SOM was calculated according to the following equation [38]:
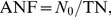
(3)where *N_o_* is the potential mineralized N as 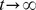 (mg kg^−1^), and TN is initial total nitrogen.

### Statistical analysis

Nitrogen mineralization data were transformed using a square root transformation to ensure normality and homogeneity of the variance. Three-way ANOVA was used to test the effects of incubation time, temperature, salinity and their interactions on nitrogen mineralization. Non-linear regression analysis was implemented to determine model parameters. Statistical analyses were conducted using the SPSS 13.0 software package.

## Results

### Nitrogen mineralization process

**Figure 1:**
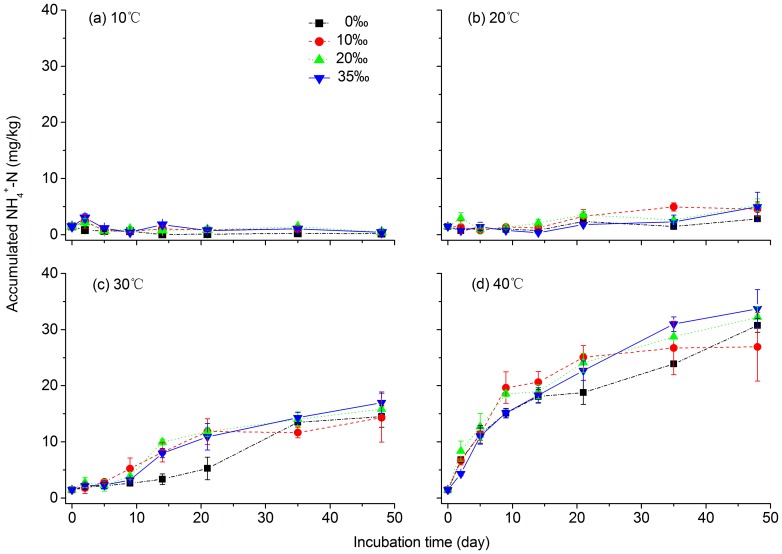
Soil accumulated ammonium nitrogen over 48 days of laboratory incubation under four temperature and salinity levels.

Figure 1 shows the accumulated soil NH_4_^+^-N over a 48-d incubation period for four salinity levels and temperature. In general, the accumulated NH_4_^+^-N ranged from 0.05 to 33.74 mg kg^−1^ and was generally significantly increased with increasing incubation time for temperatures of 20°C, 30°C and 40°C (*r* ^2^ = 0.77, *P*<0.001, Table 2 and Figure 1). ANOVA analysis showed that the incubation time significantly affected N mineralization rates (*P*<0.001). However, a decreasing trend was found at 10°C, irrespective of the salinity concentrations (Figure 1). The incubation temperature also exhibited a significant effect on N mineralization in this study (*P*<0.001, Table 2). The accumulated NH_4_^+^-N under incubation temperatures was ranked as follows: 40°C>30°C>20°C>10°C, regardless of the chosen salinity. Significantly higher mineralized N was observed at higher temperatures. It is noted that a much lower level of the accumulated NH_4_^+^-N was maintained over the incubation time under four salinity levels at 10°C. By the end of the 48-d incubation period, the accumulated NH_4_^+^-N at 40°C was two and five times higher than at 30°C and 20°C, respectively. Meanwhile, the accumulated NH_4_^+^-N for a given temperature remained nearly the same during the entire incubation period under all salinity treatments. One exception was found for a temperature of 40°C after 10 days.

**Table 2:** Results of three-way ANOVA on the soil accumulated N mineralization.

Source	df	*F*	*P*
Salinity	3	19.2	< 0.001
Temperature	3	2686.3	< 0.001
Time	6	282.5	< 0.001
Salinity × Temperature	9	1.9	> 0.05
Salinity × Time	18	3.6	< 0.001
Temperature × Time	18	90.9	< 0.001
Salinity × Temperature × Time	54	2.1	< 0.001
Error	178		

The accumulated NH_4_^+^-N was lower before 10 days into the incubation period at 20°C, 30°C and 40°C under 35‰ salinity concentration compared to the other salinity treatments. As shown in Table 2, salinity also showed significant effects on the mineralized N (*P*<0.001). The accumulated NH_4_^+^-N was generally higher at salinities of 20‰ and 35‰ compared with salinities 0‰ and 10‰ (Figure 1). In particular, the accumulated NH_4_^+^-N under 0‰ salinity treatment showed significantly lower values after 10 days of incubation at 30°C and 40°C than other salinity treatment (*P*<0.05). Incubation time, temperature, salinity and their interaction effects on N mineralization were all significant (*P*<0.001, Table 2), except for the salinity and temperature interaction (*P*>0.05, Table 2). Generally, temperature exhibited the greatest effect on N mineralization according to the F values, followed by the incubation time, whereas salinity exhibited the least effects on N mineralization (Table 2).

### Nitrogen mineralization modeling

**Figure 2:**
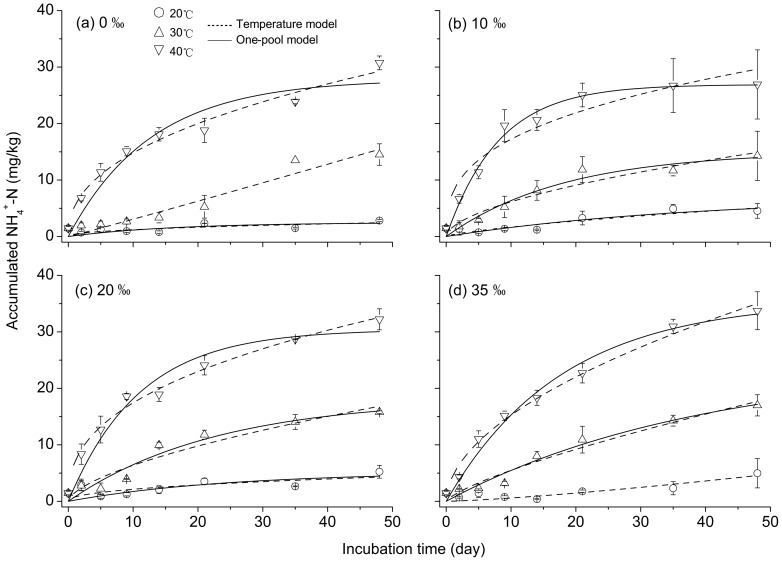
Comparisons of predicted (solid lines) and observed (symbols with dashed lines) cumulative mineral-N over 48 days of incubation under four temperature and salinity levels.

The accumulated NH_4_^+^-N at 10°C was not simulated using the temperature and one-pool models because it remained nearly stable over the 48-d incubation period (Figure 2a). The parameters (*k*, *n*) for both models were identified based on the experiment data under other incubation temperatures, i.e., 20, 30 and 40°C (Tables 3 and 4). Generally, *k* ranged from 0.004 to 2.552 and increased with increasing incubation temperature and *n* ranged from 0.346 to 1.299 and decreased with increasing temperature for the temperature model (Table 3). Moreover, *k* was highest at 0‰ salinity and 40°C and lowest at 35‰ salinity and 20°C; *n* was highest at 35‰ salinity and 20°C and lowest at 10‰ salinity and 40°C. In addition, no pronounced changing trends were observed between salinity concentrations at the same incubation temperature for both *k* and *n*. Both the effective accumulated temperature and one-pool models fit well with the predicted N mineralization under higher temperatures of 30 and 40°C (Figure 2). The regression analyses (Tables 3 and 4) showed that these parameters were ideal for both models with a better fitness (*R* ^2^ = 0.88–0.99, *P*<0.01) at both 30 and 40°C than at 20°C (*R* ^2^ = 0.30–0.85, *P*<0.05).

**Table 3:** Parameter values used for the effective accumulated temperature model over 48 days of waterlogged incubation experiments.

Salt added, ‰	20°C			30°C			40°C		
	*k*	*n*	*R^2^*	*k*	*n*	*R^2^*	*k*	*n*	*R^2^*
0	0.029±0.187	0.449±0.183	0.604	0.017±0.019	1.013±0.174	0.936	1.344±0.326	0.434±0.037	0.974
10	0.106±0.095	0.705±0.176	0.839	0.366±0.204	0.563±0.092	0.927	2.552±1.061	0.346±0.065	0.889
20	0.371±0.402	0.447±0.221	0.449	0.287±0.196	0.619±0.112	0.914	1.931±0.307	0.398±0.025	0.986
35	0.004±0.008	1.299±0.392	0.808	0.130±0.072	0.746±0.090	0.963	0.817±0.185	0.530±0.035	0.987

**Table 4:** Parameter values used for the one-pool model over 48 days of waterlogged incubation experiments.

Salt added, ‰	20°C			30°C			40°C		
	*N_0_* (mg kg^−1^ soil)	*k* (day^−1^)	*R^2^*	*N_0_* (mg kg^−1^ soil)	*k* (day^−1^)	*R^2^*	*N_0_* (mg kg^−1^ soil)	*k* (day^−1^)	*R^2^*
0	2.483±0.776	0.061±0.045	0.497	−	−	−	27.826±2.870	0.078±0.021	0.880
10	6.906±3.277	0.027±0.020	0.846	15.033±1.378	0.055±0.011	0.965	26.908±0.792	0.123±0.012	0.983
20	5.181±2.875	0.041±0.044	0.302	18.201±2.795	0.044±0.014	0.944	30.417±2.17	0.091±0.018	0.923
35	−	−	−	24.332±5.079	0.025±0.008	0.974	35.569±2.117	0.056±0.008	0.982

− no fitting parameter.

In general, *N*_0_ and *k* increased with increasing incubation temperature for the one-pool model (Table 4). Moreover, *N*_0_ increased and *k* decreased with increasing salinity (i.e., from 10 to 35‰). At 20°C and 40°C, *N*_0_ was lower than the observed accumulated NH_4_^+^-N at the end of the incubation period; the opposite was true at 30°C (Figure 2; Table 4). The ANF increased with increasing incubation temperature (Table 5). The potential mineralized N from SOM ranged from 0.65% to 9.36% of total N (0.38 g/kg, Table 1) under the four salinity treatments. The incubation times required to mineralize half of the *N_0_* (*t_1/2_*) were generally less than 2 weeks and longest for 4 weeks. Additionally, a rapid decrease in *t_1/2_* was observed from 30°C to 40°C under 35‰ salinity compared with the other salinity treatments. Compared with the temperature model over the entire 48-d incubation period, the one-pool model better represented the observed accumulated NH_4_^+^-N values at 30°C for all the four salinity concentrations.

**Table 5:** Estimates of active N fraction (ANF) and time required to mineralize half of the *N_0_* (*t_1/2_*) using the one-pool model.

Salt added, ‰	20°C		30°C		40°C	
	ANF (%)	*t_1/2_* (d)	ANF (%)	*t_1/2_* (d)	ANF (%)	*t_1/2_* (d)
0	0.65	11.4	-	-	7.32	8.9
10	1.82	25.7	3.96	12.6	7.08	5.6
20	1.36	16.9	4.79	15.8	8.00	7.6
35	-	-	6.40	27.7	9.36	12.4

## Discussion

### Effects of temperature and salinity on N mineralization

The accumulated NH_4_^+^-N increased with increasing incubation time under almost all temperature treatments except at 10°C (Figures 1 and 2). This finding is consistent with results of previous studies in wetland and paddy soils [17], [33], [34], [37]. The reason for the significant effect of incubation temperature on N mineralization in this study (*P*<0.01, Table 2) was that because temperature could greatly affect soil microbial processes [6], [10], [16], [29], [39] and higher temperature favored microbial and enzyme activity [2], [40] and thus enhanced N mineralization processes. In contrast, lower temperatures (e.g. 10°C) exhibited inhibiting effects (Figure 1) due to lower microbial and enzyme activities [19], [41]. Grenon et al. [42] also showed that most microbial metabolic rates are positively related to temperature. However, at extremely lower temperature (e.g. freezing) in non-growing seasons, higher N mineralization rates might occur due to lower N immobilization of microbes [2], [19]. Root mortality at freezing temperature might enhance N immobilization sine more available C would be released from detritus and enhance microbial activity [19]. Moreover, lower microbial biomass was likely correlated with faster net N mineralization [18], [19]. Therefore, microbial activities can be used to predict the N mineralization rate because soil microorganisms are primarily responsible for the transformation of organic N to mineral N [10], [34].

Studies have reported that N mineralization decreased with increasing salinity and was biologically mediated at higher salinities [12], [20], [23] because microbial activity can be inhibited at high salinities (>500 mS m^−1^) [6], [29], [43]. Muhammad et al. [44] and Tripathi et al. [45] also presented that both soil respiration and soil microbial biomass were negatively correlated with soil salinity. Additionally, Wong et al. [46] addressed that high salinity (30 dS m^−1^) increased electrolyte concentrations and caused the soil to flocculate, preventing the organic N release. However, obvious inhibiting effects of high salinity on N mineralization processes were not observed in this study. A higher accumulated NH_4_^+^-N corresponded with higher salinity treatments, which was supported by Neo et al. [17], who suggested that increasing salinity from 0.1 to 3.5‰ contributes to soil N mineralization in tidal forested wetlands. Chen and Twilley [22] also observed higher N mineralization with increasing salinity in mangrove soils. The ANOVA analysis further showed significant effects of salinity on the accumulated NH_4_^+^-N (*P*<0.001, Table 2). However, the lower accumulated NH_4_^+^-N in the first 10 incubation days before its increase under 35‰ salinity compared to the other salinity treatments might imply a short-term inhibiting effect before enhancing NH_4_^+^-N release. Khoi et al. [47] also observed that adverse effects of salinity on N mineralization were short-lived, whereas the rate of N mineralization recovered in later stages. Further studies are needed to testify this effect in different wetland soils under different vegetation.

Generally, the effects of increasing salinity on N mineralization can be explained as follows. Firstly, under high salinity conditions, a shift in microbial population structure occurs [46]. Muhammad et al. [44] found higher proportions of fungal biomass in more saline soils compared with less saline soils. Therefore, the soil ammonification process can be enhanced as an effect of salinity on specific microbial function groups responsible for ammonification, which results in increased accumulated NH_4_^+^-N at higher salinities (Figure 1). Secondly, Na^+^ is an important and dominant seawater component. High electrical conductivity solutions, particularly those high in Na^+^, could rapidly alter the composition of exchange sites on clays, causing NH_4_^+^ adsorbed on to clay surfaces to be desorbed [46], leading to the increase in mineralized N. Thirdly, the inhibiting effects of salinity on N mineralization processes might be mitigated by high temperatures [29], because N mineralization was largely improved at higher temperatures (i.e., 30°C and 40°C, Figure 2). Meanwhile, the ANOVA analysis also showed a significant effect of temperature (larger than the salinity effect) on the accumulated NH_4_^+^-N (*P*<0.001, Table 2). Although no significantly interactive effect of temperature and salinity was observed, (*P*>0.05, Table 2), the significant interactive effect was displayed (*P*<0.001, Table 2) if incubation time was further took into account. This indicated that incubation time was also an important factor influencing the accumulated NH_4_^+^-N [4]. Therefore, long-term incubation experiments are required to better understand the soil N mineralization process.

### Nitrogen mineralization models

It is well known that mineralization is a process in which soil organic compounds are decomposed by microorganisms into inorganic compounds. Moreover, microbial mineralization activity depends on enzyme kinetics, which follows a first-order dynamical model [48]. Models and their corresponding parameters are essential to mathematically describe soil N mineralization kinetics [49]. The first-order reaction kinetic model was firstly used by Stanford and Smith to simulate aerobic long-term mineralization dynamics [37], while other exponential models have been used for N mineralization in recent studies [6], [48], [50].In this study, the ideal parameters for both the effective accumulated temperature and one-pool models indicated that both models could simulate N mineralization in tidal freshwater wetlands at higher temperatures of 30 and 40°C (Figure 2). However, a better simulation was observed at 40°C for the temperature model, whereas a similar performance was obtained between the temperature and one-pool models at less than 40°C, which is consistent with results of previous studies [6], [38], [50].

The effective accumulated temperature model, which is regarded as the empirical formula derived from the first-order reaction kinetic model, explains the enzyme kinetics. In general, the mineralization constant (*n*) for the temperature model should be between 0 and 1 based on the theory of enzyme kinetics [33], [38]. Moreover, *n* decreased with increasing temperature, suggesting that mineralized N per effective accumulated temperature decreased with increasing effective accumulated temperature due to higher enzyme activity [38]. However, a higher *n* was observed for either lower or higher salinities in this study (Table 2). The *k* value reflects the mineral extent of mineralization [47]. Compared with lower temperature (20°C), the larger *k* values at higher temperature exhibited higher mineral extent of mineralization, indicating temperature is a vital factor of N mineralization. Bai et al. [33] and Li et al. [48] also demonstrated that the effective accumulated temperature model (temperature model) was a N mineralization model with temperature as the dominant factor. Bai et al. [33] also testified that a temperature model can efficiently simulate N mineralization in inland salt marshes. Therefore, the temperature model can be used to describe the N mineralization process when the temperature exhibits substantial effects on N mineralization.

For the one-pool model, *k* (day^−1^) is considered as the percentage of the remaining mineral N that is mineralized per day [37], which decreased with increasing salinity. In contrast, *N*_0_ generally increased with increasing salinity, implying that a higher N mineralization potential existed under higher salinity conditions. Additionally, the ratio of potential mineralized N (*N_0_*) to total N represents the active N fraction (ANF) in the SOM. The general increasing of ANF and decreasing of incubation time to mineralize half of *N_0_* (*t_1/2_*) corresponded with increasing incubation temperature (Table 5), which again indicated that increasing temperature could substantially accelerate N mineralization. The time of *t_1/2_* for all salinity treatments ranged from > 10 days to < 10 days from 20°C to 40°C, indicating a quick mineralization at higher temperatures. Meanwhile, smaller *t_1/2_* reflected large value of the mineralization rate constant (*k*) (Table 5) and indicated that the small ANF of a soil was easily mineralized and readily used by microorganisms [38]. However, compared with other studies [38], [48], *N_0_* and ANF were much smaller at 20°C and 30°C (Table 5). Although the incubation time was not as long (12 to 40 weeks) as in other studies [33], [49]–[51], all mineralization half times under all treatments were within a 48-d incubation period (Table 5). This time frame reflected the N mineralization process in the N-limiting tidal freshwater wetland soils. Furthermore, the rapid decrease in *t*_1/2_ from 30°C to 40°C was observed under 35‰ salinity than under other salinity treatments (Table 5), indicating that a higher salinity might contribute to an enhanced N mineralization. However, the short-term inhibiting effects of 35‰ salinity on the N mineralization rate might also occur at higher temperature of 40°C in the first 10 incubation days (Figures 1 and 2) due to lower enzyme activity [2] and higher N immobilization of microbes [2], [19].

Theoretically, *N_0_* should be greater than the observed cumulative mineral N over the incubation period [48], [49] because N_0_ is defined as the entire pool of potentially mineralizable N. However, the potential mineralized N at 20°C and 40°C from the one-pool model were very close to or even smaller than the observed mineralized N (Table 4). Nevertheless, the one-pool model had a better fit to the observed data than the double first-order exponential model (two-pool model) [52], which was used to simulate the accumulated NH_4_^+^-N over the incubation period. Moreover, the two-pool model did not fit the observations well and contained large standard errors (data not shown) This finding contradicts the results of Li et al. [48], who concluded that a two-pool model was better for determining accumulated mineralizable N in paddy soils. However, Cordovil et al. [38] reported that the two-pool model had no advantage to fit the experimental data over one-pool model as it could not identify materials with different mineralization rate kinetics. Stanford and Smith [37] reported that the pool containing the compounds that contribute to the potentially mineralizable N was similar in most soils. Therefore, only one pool is often required [38].

## Conclusions

NH_4_^+^-N release was significantly enhanced with increasing temperature and salinity over a 48-d incubation period. Moreover, accumulated NH_4_^+^-N was significantly higher under higher salinity than under lower salinity levels at higher temperatures. Except for the interaction of temperature and salinity, incubation time, temperature and salinity and their interactive effects exhibited significant effects on N mineralization; and temperature dominated the effects. Accumulated NH_4_^+^-N increased with increasing incubation time, which fit well with the simulation by both the effective accumulated temperature and one-pool models at temperatures up to 40°C. Although we do not compare N mineralization among different soil types overlaying vegetation in this study, our results indicated that NH_4_^+^-N release was increased in tidal freshwater wetland soils after the conversion to brackish wetlands due to sea-level rise, which might occur as a result of projected global warming. Therefore, the soil N mineralization response to increasing temperature and salinity in tidal freshwater wetlands should be integrated with studies on the structure and functions of coastal ecosystems in response to global warming and sea-level rise. Additionally, further investigation is needed to reveal the effects of salt anions (e.g. Cl^−^, SO_4_^2−^) and on N mineralization in wetland soils. The combined study on N mineralization though lab and *in situ* incubation experiments should be focused on in the future.
